# Draft Genome Sequence of Polaromonas eurypsychrophila AER18D-145, Isolated from a Uranium Tailings Management Facility in Northern Saskatchewan, Canada

**DOI:** 10.1128/mra.00013-22

**Published:** 2022-03-09

**Authors:** Alexander A. Grigoryan, Viorica F. Bondici, Yuriy Kryachko, Nurul H. Khan, John R. Lawrence, Gideon M. Wolfaardt, Niradha Withana Gamage, Deeksha Shetty, Darren R. Korber

**Affiliations:** a Food and Bioproduct Sciences, University of Saskatchewan, Saskatoon, SK, Canada; b Saudi Aramco, Dhahran, Saudi Arabia; c Canadian Light Source, Saskatoon, SK, Canada; d Lallemand Plant Care, Saskatoon, SK, Canada; e Environment Canada, Saskatoon, SK, Canada; f Department of Chemistry and Biology, Ryerson University, Toronto, ON, Canada; g Department of Microbiology, Stellenbosch University, Stellenbosch, South Africa; University of Southern California

## Abstract

The 4.8-Mbp draft genome sequence of Polaromonas eurypsychrophila AER18D-145, isolated from a uranium tailings management facility, is reported. The sequence may provide insights into the mechanisms of the hypertolerance of this strain to extreme conditions and help determine its potential for bioremediation applications.

## ANNOUNCEMENT

*Polaromonas* spp. have been reported to be among the most abundant microorganisms in glacial and seasonally cold nonglacial environments (1–5). These bacteria were shown to be capable of oxidizing molecular hydrogen (6), arsenite (7), and various recalcitrant organic compounds (8, 9). Several *Polaromonas* species were demonstrated to be capable of nitrate reduction (10, 11). Sun et al. (12) suggested that some *Polaromonas* spp. might also be capable of vanadate reduction. Despite the fact that several microorganisms belonging to this genus have been previously isolated and their metabolic capabilities investigated, few studies have been dedicated to the determination of genome sequences of *Polaromonas* spp. inhabiting uranium-rich environments.

Here, we report the draft genome sequence of Polaromonas eurypsychrophila AER18D-145 from a uranium tailings management facility in Key Lake, Northern Saskatchewan, Canada (57°13′N, 105°38′W). The strain was isolated from a tailings sample collected at an 18-m depth below the tailings-water interface (13). To isolate the microorganism, 0.2 g of the sample was suspended in 1 mL of sterile Tris-EDTA buffer, pH 8, plated on Reasoner’s 2A (R2A) agar, and incubated aerobically at 5°C for 3 weeks. Following isolation, colonies were subcultured three times. The pure culture was stored at −80°C in 15% glycerol/5% tryptic soy broth. A DNA extraction kit (Qiagen, Maryland, USA) was used to extract DNA from glycerol-stock cells, which were regrown on R2A agar.

Genomic DNA was extracted using the DNeasy blood and tissue kit (Qiagen) according to the manufacturer’s recommendations. Libraries were prepared using the Nextera XT library preparation kit (Illumina) with a MiSeq reagent 300-cycle V2 kit (Illumina), and sequencing was performed on an Illumina MiSeq instrument, resulting in 725,002 paired reads (209.75 Mbp). The A5-miseq assembly pipeline version 20140604 (14, 15) was used for error correction, quality trimming, contig assembly, misassembly corrections, and scaffolding. The genome consists of 135 contigs (*N*_50_, 77,773 bp) and is 4,822,403 bp long; no gaps were identified. The genome coverage is 42×, and the G+C content is 63.1%. Annotation of the genome was done using the NCBI Prokaryotic Genome Annotation Pipeline (PGAP) version 5.1 (16, 17). As a result of annotation, 4,490 protein-coding sequences, as well as 49 RNA-coding sequences, were identified in the genome.

**FIG 1:**
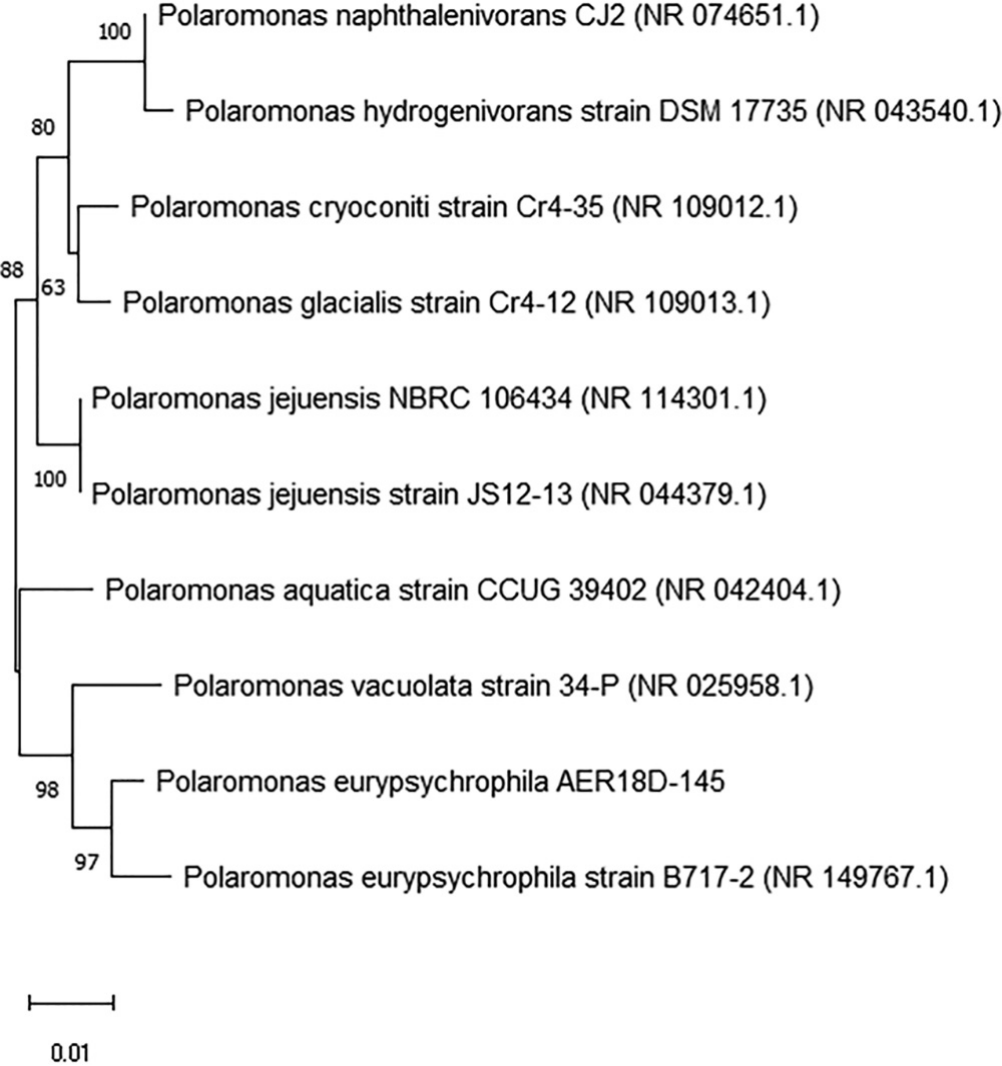
Neighbor-joining tree showing type strains having the highest percent identities to *P. eurypsychrophila* AER18D-145, as determined through the comparative analysis of 16S rRNA gene sequences. GenBank accession numbers of the type strains are shown in parentheses. Bootstrap values are indicated next to the tree. The scale bar indicates the number of nucleotide substitutions per site. The tree was generated using MEGA version 11 (22); the MUSCLE sequence alignment method (23), 1,000 bootstrap replications, and the maximum composite likelihood nucleotide substitution model/method were selected; otherwise, MEGA default settings were applied for phylogeny reconstruction.

Comparison of the 16S rRNA gene sequence of *P. eurypsychrophila* AER18D-145 to the RefSeq database sequences (18) using the BLASTN algorithm (19) showed that its 1,425-bp fragment was 100% identical to that of *P. eurypsychrophila* strain D3M1 (GenBank accession number MW647764). Comparison to the 16S rRNA gene sequences of type strains indicated the highest percent identity of 98.8% to *P. eurypsychrophila* strain B717-2 (11) (Fig. 1), confirming the species identity of *P*. *eurypsychrophila* AER18D-145.

Some genes indicating the potential utility of this bacterium in bioremediation applications were identified through the genome analysis using RAST (Rapid Annotations using Subsystems Technology) version 2.0 (20, 21). In particular, *merA*, *merP*, and *merT*, responsible for mercury resistance, *chrA* and *chrF*, responsible for resistance to chromium-containing compounds, and *dedA* and *cysA*, which may play roles in selenium oxyanion uptake, were among the identified genes.

### Data availability.

This whole-genome shotgun project was deposited in DDBJ/ENA/GenBank under the accession number NZ_NBZV00000000. The raw data were deposited in the SRA under the accession number SRR16891862 (BioProject number PRJNA381359).
